# Interactions between Pre-Emergence Herbicides and Nematicides on the Soybean Growth and Nematode Population

**DOI:** 10.1021/acsomega.5c08857

**Published:** 2025-12-04

**Authors:** João Guilherme Queiroz Bordignon, Pedro Delefrate Neto, Hércules Diniz Campos, Laís Tereza Rego Torquato Reginaldo, Camila Rebelatto Muniz, Guilherme Braga Pereira Braz, Matheus de Freitas Souza

**Affiliations:** 1 245072 Universidade de Rio Verde, Rio Verde 75901-970, Brazil; 2 Agronomy College, Universidade de Rio Verde, Rio Verde 75901-970, Brazil; 3 5547 Mississippi State University, Starkville 39762, United States of America

## Abstract

In recent years, the combined pressure from nematodes and weeds has increased considerably. Consequently, the spray of mixed nematicides and pre-emergence herbicides has become a common practice in soybean fields. However, the interactions between these products are poorly understood. We hypothesize that additive, synergistic, or antagonistic effects may occur due to their simultaneous application. Therefore, this study aimed to investigate the interactions between nematicides and herbicides applied at soybean pre-emergence on the control of *Pratylenchus brachyurus* populations and the initial development of soybean plants. Three independent experiments were conducted, each corresponding to one nematicide treatment, and each experiment was repeated twice under similar field conditions. All experiments were carried out in a greenhouse under a 2 × 5 + 2 factorial design. The first factor consisted of the presence or absence of a nematicide. The second factor included five pre-emergence herbicides. Two additional control treatments were also included. Biometric variables of soybean plants and nematode population densities were evaluated. The results indicated that the combination of [flumioxazin + imazethapyr] increased nematode populations, whereas [flumioxazin + *S*-metolachlor] and [fomesafen + *S*-metolachlor] reduced nematode populations and enhanced soybean root biomass accumulation. The [flumioxazin + imazethapyr] mixture negatively affected the nematicidal activity of *Bacillus amyloliquefaciens* BV03, both when applied as a seed treatment and as a foliar spray. In contrast, the antagonistic effect of [flumioxazin + pyroxasulfone] on *B. amyloliquefaciens* BV03 was observed only when the nematicide was applied as a seed treatment. Some herbicides reduced *P. brachyurus* populations independently of nematicide presence, while others showed additive effects when combined with the nematicide. The interactions observed in this study underscore the importance of carefully selecting products within an integrated management context, considering not only their individual efficacy but also their combined effects on soil and plant health.

## Graphical abstract

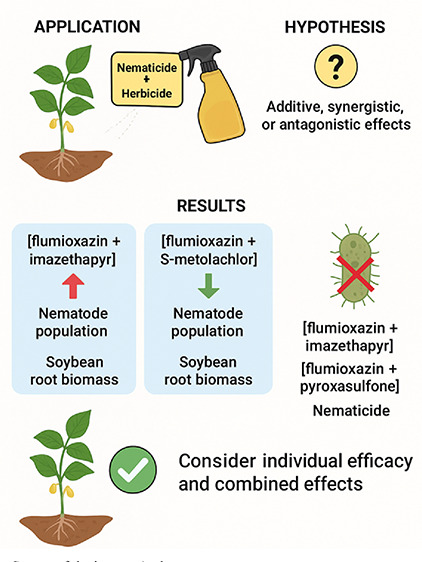

## Introduction

Soybean is one of the most important agricultural crops worldwide, playing a key role in food security and the production of more sustainable fuels. In terms of food security, its high content of high-quality protein and oil makes soybean an affordable alternative to meat, especially in low-income regions.^1^ However, soybean production is often hindered by phytosanitary problems, such as weeds and nematodes. Weeds can cause significant yield losses in soybean crops when improperly managed.^2^ For example, delaying weed control from 14 to 42 days after sowing may result in yield losses of approximately 32 to 37 kg ha^–1^ for each day of interference.^3^ Furthermore, in recent years, weed management has become increasingly challenging due to the evolution of herbicide-resistant biotypes, especially those resistant to postemergence herbicides like glyphosate.^4^

Weed resistance to herbicides represents a major challenge for agriculture, with 253 resistant species reported worldwide and annual losses reaching 33 billion dollars in the United States.^5^ In Australia and India, the economic impact can reach annual losses estimated at 3.3 billion Australian dollars and over 11 billion dollars, respectively.^5^ In Brazil, data on monetary losses are unclear, but losses in soybean yield can reach up to 30%, mainly due to infestations by *Pratylenchus brachyurus*.^6^ Among the recommended practices for the integrated management of weeds resistant to glyphosate and other postemergence herbicides used in soybean, such as clethodim and imazethapyr, the use of pre-emergence herbicides has enabled the adoption of a broader range of modes of action within the production system, delaying the spread of resistant biotypes and tolerant species.^7^

Another common phytosanitary problem in soybean fields is nematodes. These phytopathogenic organisms can infect soybean roots, causing direct damage that compromises nutrient uptake, as well as biochemical and physiological changes capable of affecting plant growth and development and/or favoring the occurrence of disease complexes.^8,9^ This set of negative effects, especially under the high population pressure of the pathogen, leads to considerable yield reductions if no mitigation measures are adopted by the grower. The main nematodes affecting soybean crops are the soybean cyst nematode (*Heterodera glycines*), the root-knot nematode (*Meloidogyne incognita*), and root lesion nematodes (*Pratylenchus* spp.), which are responsible for significant yield losses in several producing regions.^7^ For root lesion nematodes, soybean yield losses can reach up to 30% of the potential yield.^8^

Given the losses caused by nematodes, controlling the spread of nematode populations is a crucial step in soybean production. Nematode management in soybean crops requires the adoption of strategies that combine different control tactics, with the focus on reducing populations and mitigating production losses. Cultural practices such as rotation with nonhost species, the use of resistant cultivars, and quarantine and sanitation actions have proven effective in breaking the nematode cycle and preventing their spread.^10^ However, when these practices are not enough to keep population levels low, biological and chemical methods become an important tool. Biological control, for example, has gained prominence due to the use of agents such as *Bacillus subtilis* and *Purpureocillium lilacinum*.^11^ The main advantages of these alternatives are their selectivity, low environmental impact, and contribution to the sustainability of the production system. Chemical nematicides have also been important when other methods are not sufficient, representing an effective measure in situations of high population pressure and immediate need for control.^12^

In recent harvests, soybean producers have observed an increase in both the incidences of weeds and nematodes. For weeds, the main reason behind the rise for some species has been the spread of weed-resistant biotypes to glyphosate and other herbicides applied in the postemergence. For nematodes, the intensification of production systems focused on the recurrent use of host crops in succession and reduced soil preparation (or absence in no-until areas) has contributed to the accumulation of inoculum and the dissemination of nematodes, such as *Pratylenchus* spp. Consequently, the application of chemical or biological nematicides and herbicides has been carried out in areas cultivated with soybeans, oftentimes close to the crop cycle. For example, many of the nematicides are used via seed treatment or in-furrow application at the time of planting. In scenarios where the producer will apply herbicides in pre-emergence, generally 1 or 2 days after planting, nematicides and herbicides will share the same environment in the soil. Therefore, it is necessary to understand which interactions may occur between these products to ensure the effective recommendation of these control methods.

Studies have already shown that herbicides and nematicides may exhibit distinct interactions with each other, resulting in synergistic, additive, and antagonistic effects.^13,14^ Among these possibilities, antagonistic effects are critical because they may reduce the effectiveness of the active ingredient in controlling the target. In this context, we hypothesize that additive, synergistic, or antagonistic effects may occur due to their simultaneous application. Therefore, this study aimed to investigate the interactions between nematicides and herbicides applied at soybean pre-emergence on the control of *P. brachyurus* populations and the initial development of soybean plants.

## Material and Methods

### Design Experimental

The experiment was conducted twice in a greenhouse to ensure repeatability of the results. The joint analysis of the trials is described below. Each trial was carried out in a completely randomized design with three replications. Two nematicides and five herbicides were selected for the experiments. Two controls—one without nematicides and herbicides and one with only nematicides (applied according to each treatment)—were added to the set of treatments, resulting in a 2 × 5 + 2 factorial arrangement.

The nematicides and herbicides used in this experiment were selected based on their performance and frequency of use in commercial soybean fields. The previous performance of the nematicides was obtained from the published literature to ensure that the tested nematicides had proven efficacy in nematode control. The herbicides were selected following the same criteria as those for the nematicides. The herbicides used were [flumioxazin + *S*-metolachlor], *S*-metolachlor, [fomesafen + *S*-metolachlor], [flumioxazin + pyroxasulfone], and [flumioxazin + imazethapyr]. The nematicides used were *Bacillus amyloliquefaciens* BV03 (No-Nema, Vittia, Brazil), applied as seed treatment and foliar application on soybean, and fluopyram (Verango Prime, Bayer Crop Science, Brazil). Compound information used is shown in the Supporting Information.

### General Information

5 dm^3^ pots were filled with a substrate containing a medium-textured soil (clay:sand ratio). After filling and irrigating the pots to field capacity,^15^ three seeds of soybean, cultivar Taquari HO (supplied by SEEDCORP|HO/HO Genética), were sown in each pot. During sowing, the treatments containing nematicides recommended for seed treatment were applied at the doses recommended by the commercial label (doses mentioned below).

One nematicide was applied via seed treatment (BV03-ST), one in the planting furrow (fluopyram), and a third nematicide treatment was applied at the V4 phenological stage of soybean, approximately 20 days after crop emergence (DAE), (BV03-FA), also following the label-recommended doses. For the application of *Bacillus amyloliquefaciens* BV03 in seed treatment, a dose of 3 mL kg^–1^ of seed was used (with a spray volume of 6 mL kg^–1^ to ensure better distribution of the product around the seed). For fluopyram application, a dose of 0.5 L ha^–1^ was used (with a spray volume of 80 L ha^–1^). The foliar application of *B. amyloliquefaciens* BV03 was performed at a dose of 1 L ha^–1^ (with a spray volume of 200 L ha^–1^).

The herbicides were applied 2 days after planting for pre-emergence weed control. The doses used followed the recommendations on the commercial product labels as follows: Apresa (flumioxazin 42 g ha^–1^ + *S*-metolachlor 840 g ha^–1^; ADAMA, Brazil), Dual Gold (*S*-metolachlor 1,440 g ha^–1^; Syngenta, Brazil), Eddus (fomesafen 170.8 g a.e. ha^–1^ + *S*-metolachlor 776.8 g ha^–1^; Syngenta, Brazil), Kyojin (flumioxazin 60 g ha^–1^ + pyroxasulfone 90 g ha^–1^; IHARA, Brazil), and ZethaMaxx (imazethapyr 80 g a.e. ha^–1^ + flumioxazin 40 g ha^–1^; Sumitomo Chemical, Brazil). All applications were performed with a spray volume of 150 L of ha^–1^.

Applications were made using a CO_2_-pressurized backpack sprayer (Herbicat, Catanduva, Brazil) at a pressure of 2.5 bar. The spraying system had a two-nozzle boom with flat fan nozzles (Teejet XR—″fan″ type) 110.02, spaced 0.5 m apart. The application rate varied depending on the treatment described above. The application conditions followed the recommended parameters for pesticide application, with relative humidity around 60%, air temperature at 25 ± 5 °C, and maximum wind speed of 2.5 km h^–1^. Both the pre-emergence herbicide and postemergence nematicide applications were performed according to the instructions described above.

A solution containing 1 mL of 1000 viable *P. brachyurus* nematodes was used as an inoculum in treatments involving nematodes. This solution was added to the pots after soybean emergence. The solution was prepared from a prior nematode collection in an area with a nematode history. This area is a commercial field used for soybean, corn, and sorghum production. The last three growing seasons in this area consisted of soybean–corn succession (2021/2022), soybean–sorghum (2022/2023), and soybean–sorghum (2023/2024). The geographic coordinates of the site are 17°47′18″S, 50°57′31″W. At the time of collection, the field was cultivated with sorghum (cv. G100 [Agroceres Seeds, Brazil]). Consequently, nematode extraction was performed from sorghum roots because *P. brachyurus* is also a known host species of this crop. Sorghum was grown in the V8–V10 stages. Sorghum roots were collected and blended in water. After blending, the solution was sieved to remove root debris. Subsamples of this solution were used for viable nematode counts to ensure the concentration of 1000 nematodes per 1 mL of solution.

### Variables Evaluated

The evaluation of the experiments always began after the application of the BV03-PA treatment, that is, when the soybean plants reached the V4 stage. For both trials, the BV03-PA treatment was applied at 20 DAE. From that point on, the variables evaluated were plant height, stem diameter, and number of trifoliate leaves at 27, 34, 41, and 60 DAE. Shoot and root dry mass, root volume, and number of nematodes in the root system were evaluated at 60 DAE.

Plant height was measured by using a ruler, considering the distance from the soil surface to the insertion of the last fully expanded trifoliate leaf. Stem diameter was measured 1 cm above the cotyledonary node using a digital caliper (precision ± 0.02 mm). The number of trifoliate leaves was determined by counting the number of leaves per plant. For dry mass evaluation, the shoot and roots were placed in paper bags and transferred to a forced-air oven at 65 °C for 72 h. Root volume was determined by using a graduated cylinder. The root and a known volume of water were added to the cylinder. The difference between the total volume measured and the volume of water added was considered the estimated root volume (mL).

The total number of nematodes was obtained by cutting the roots into small fragments and crushing them for 30 s using a blender.^16^ Then, the *P. brachyurus* population in the suspension from each plot (treatment replicate) was quantified using a Peters counting slide under an optical microscope (Olympus CX3) at 100× magnification. After obtaining the raw counts, the number of *P. brachyurus* per gram of root was calculated.^17^

### Statistical Analysis

All statistical analyses were performed using R software version 3.6. A Pearson correlation analysis was conducted among the variables. Variables showing a high correlation with each other (>0.90) underwent a selection process, retaining only one of them. After confirming the normality of the residuals and the homoscedasticity of the variances, analysis of variance (ANOVA) was applied to the response variables obtained. An *F*-test between the variances of the first and second trials was used to assess the homogeneity of variances for all evaluated variables. Once homogeneity was confirmed, a joint analysis of the trials was performed. First, the trials were analyzed using the model: variable ∼ (1 | trial:block) + trial * [factor1 (herbicides) * factor2 (nematicides)], using the lme4 package, to verify the existence of a trial effect on the interaction factor1 * factor2. If no interaction between trial * factor1 * factor2 was detected, then a new ANOVA was carried out considering “trial” as a random variable using the following model: variable ∼ factor1 * factor2 + (1 | trial/block). When significant by the *F*-test (*p* < 0.05), the means were compared using Tukey’s test (*p* < 0.05). Dunnett’s test (*p* < 0.05) was used to compare the means of the factors with the controls “without nematicide and herbicide application” and “control with nematicide”. For non-normal variables, data transformation was applied before performing ANOVA. If non-normality persisted, then the variable was analyzed using a nonparametric test.

## Results

The Pearson correlation analysis revealed strong positive correlations (*r* > 0.90) among several plant growth variables measured at different evaluation times. High correlations were observed between plant height, stem diameter, and number of leaves measured at consecutive dates (27, 34, 41, and 60 days after emergence) as well as between root and shoot dry matter (Figure 1). The high correlations indicate that these variables describe similar patterns of plant development. Therefore, to reduce redundancy and simplify interpretation, only a subset of representative variables was retained for subsequent analyses of variance and comparison of means. The variables chosen were plant height at 60 days (alt_60), leaf number at 60 days (n_folhas_60), stem diameter at 60 days (diam_60), root dry matter (root_dry_matter), root volume (root_volume), and nematode population per 10 g of root (nema_10g).

**Figure 1:**
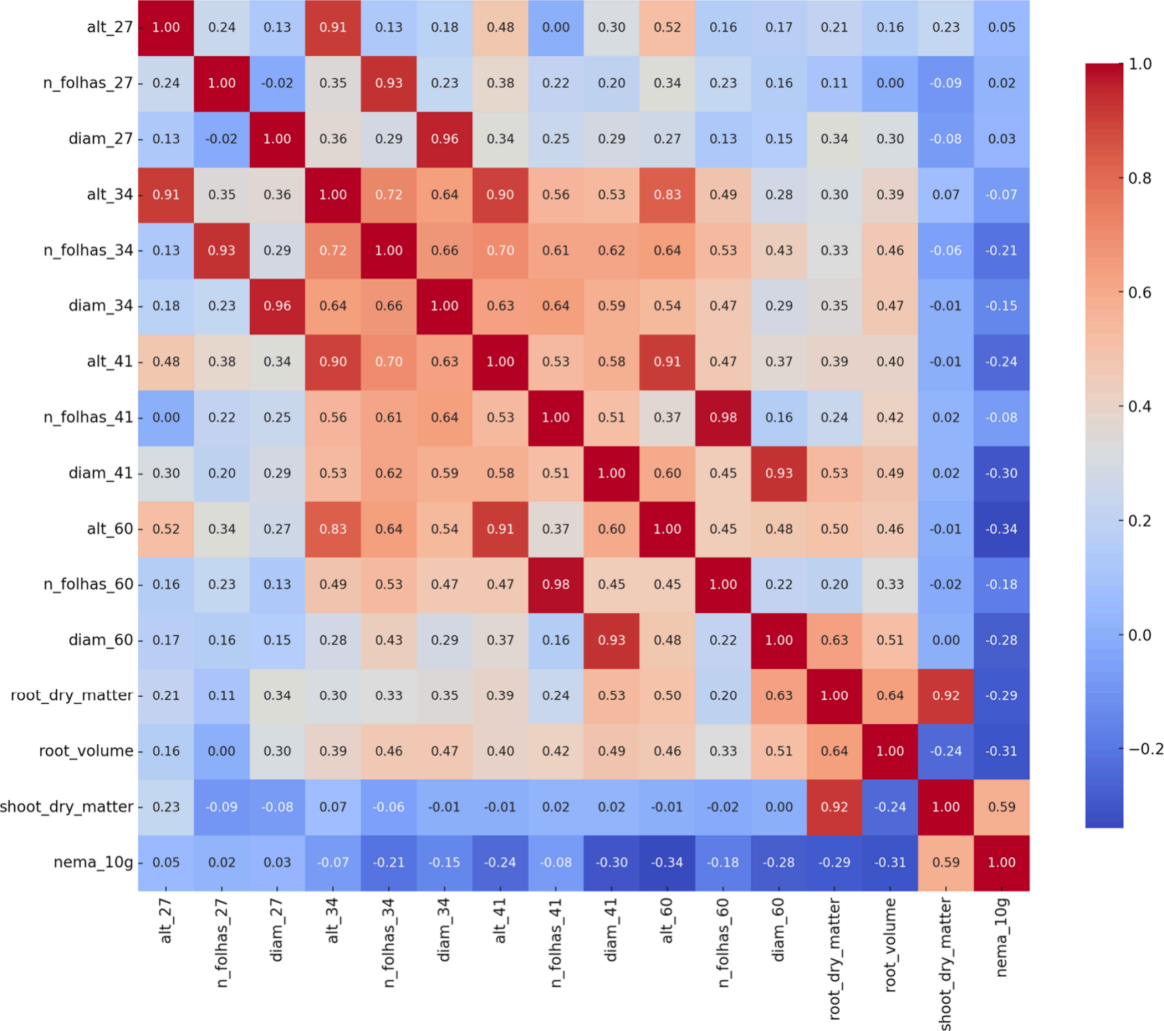
Heatmap of the Pearson correlation matrix among morphological, biometric, and nematode infestation-related variables. Colors represent the direction and magnitude of the correlation, ranging from blue (negative correlation) to red (positive correlation). The values in the cells indicate the Pearson correlation coefficients (*r*), with emphasis on pairs with *r* ≥ 0.90, which indicate high collinearity between variables.

### Interaction between Foliar Application of *Bacillus amyloliquefaciens* BV03 and Herbicides Applied in Pre-Emergence

The ANOVA results indicated that none of the evaluated variables showed a significant triple interaction among the trial, herbicide, and nematicide factors (*p* > 0.05). This outcome, based on the *F*-test from the joint analysis of variance, demonstrates that the combined effects of herbicide and nematicide treatments did not differ between the two experimental trials. In other words, the herbicide × nematicide interaction was statistically consistent across both trials, indicating similar response patterns, regardless of the experimental repetition. Therefore, the factor trial was considered a random effect in the final mixed model to account for random variability between trials while preserving the estimation of fixed effects associated with herbicides and nematicides.

Soybean plant height 60 days after emergence varied according to the herbicide treatments and the application of BV03-PA (Table 1). In general, no differences were observed among herbicides in the absence or presence of BV03-PA, ranging from 47.88 ([flumioxazin + imazethapyr]) to 52.08 cm ([flumioxazin + *S*-metolachlor]) and from 45.55 ([flumioxazin + pyroxasulfone]) to 55.45 cm ([fomesafen + *S*-metolachlor]). These treatments also did not differ from the BV03-PA control but were superior to the control without herbicide and nematicide.

**Table 1:** Plant Height (cm), Stem Diameter (cm), and Number of Leaves of Soybean Plants Subjected to Foliar Application of Nematicide (*Bacillus amyloliquefaciens* BV03–BV03-PA) and in the Presence and Absence of Herbicides Applied at Pre-Emergence

plant height (cm)—60 DAE					
	[Flum. + *S*-met.]	*S*-met.	[Fomes.+ *S*-met.]	[Flum. + pirox.]	[Flum. + imaz.]
without nematicide	52.08 aA^b^	51.83 aA^b^	51.43 aA^b^	50.88 aA^b^	47.88 aA^b^
BV03-PA	48.38 aA^b^	46.48 aA^b^	55.45 aA^b^	45.55 aA^b^	46.95 aA^b^
control with BV03-TS^a^	50.63				
control without nematicide and herbicide^b^	42.00				
CV	8.4				

Stem diameter (mm)—60 DAE					
	[Flum. + *S*-met.]	S-met.	[Fomes.+ *S*-met.]	[Flum. + pirox.]	[Flum. + imaz.]
without nematicide	5.72 aA^b^	4.89 aB	5.76 aA^b^	5.47 aA^b^	4.70 aB
BV03-PA	6.13 aA^a^ ^b^	5.03 aB	5.63 aA^b^	5.19 aB^b^	5.03 aB
control with BV03-TS^a^					
control without nematicide and herbicide^b^	5.19				
without nematicide	4.38				
CV	6.77				

Number of leaves—60 DAE					
	[Flum. + *S*-met.]	*S*-met.	[Fomes.+ *S*-met.]	[Flum. + pirox.]	[Flum. + imaz.]
without nematicide	15.00 aA	15.75 aA	13.00 aA	16.50 aA	14.25 aA
BV03-PA	13.25 aB	12.00 aA	15.25 aA	13.50 aA	15.00 aA
control with BV03-TS^a^	13.5				
control without nematicide and herbicide^b^	12.5				
CV	6.77				

a Differences between treatments and the control with BV03-PA only (BV03 applied to the foliar part) according to Dunnett’s test, *p*-value <0.05.

b Significant difference between treatments and the control without herbicide and nematicide according to Dunnett’s test, *p*-value <0.05. Lowercase letters indicate differences among treatments in the columns, and uppercase letters indicate differences in the rows, according to the Scott-Knott test, *p*-value <0.05. CV = coefficient of variation. Flum. + *S*-met. = flumioxazin + *S*-metolachlor; *S*-met. = *S*-metolachlor; Fomes. + *S*-met. = fomesafen + *S*-metolachlor; Flum. + pyrox. = flumioxazin + pyroxasulfone; Flum. + imaz. = flumioxazin + imazethapyr.

For stem diameter (Table 1), the treatments [flumioxazin + *S*-metolachlor] (6.13 mm) and [fomesafen + *S*-metolachlor] (5.63 mm) combined with the nematicide BV03-PA showed the highest means compared to the other herbicides and were superior to the control without nematicide and herbicide. In the absence of nematicide, the application of [flumioxazin + *S*-metolachlor] (5.72 mm) and [fomesafen + *S*-metolachlor] (5.76 mm) resulted in greater stem diameter compared with the other herbicides and the control without herbicide and nematicide. The other herbicides did not differ from the control without herbicide and nematicide for stem diameter, in the presence or absence of BV03-PA. For the number of leaves, no significant differences were observed among treatments and controls.

The number of nematodes per gram of root was influenced by herbicide treatments and the application of nematicide BV03-PA (Table 2). Under conditions without nematicide application, values ranged from 45.73 ([flumioxazin + *S*-metolachlor]) to 261.35 ([flumioxazin + imazethapyr]). Among these, the treatments [flumioxazin + *S*-metolachlor] (45.73), *S*-metolachlor (80.59), and [fomesafen + *S*-metolachlor] (67.97) did not differ from each other and showed lower values than the other herbicides and the control without herbicide and nematicide (155.32), indicating a reduction in nematode population even in the absence of BV03-PA. The treatment [flumioxazin + pyroxasulfone] (156.12) showed higher nematode numbers compared to the control with BV03-PA, while [flumioxazin + imazethapyr] resulted in higher nematode counts than both controls.

**Table 2:** Number of Nematodes per 10 g of Root, Root Dry Mass (g), and Root Volume (cm^3^) of Soybean Plants Subjected to Foliar Application of Nematicide (*Bacillus amyloliquefaciens* BV03–BV03-PA) and in the Presence and Absence of Herbicides Applied at Pre-Emergence

number of nematodes per 10 g of root—60 DAE					
	[Flum. + *S*-met.]	*S*-met.	[Fomes. + *S*-met.]	[Flum. + pirox.]	[Flum. + imaz.]
without nematicide	45.73 aC^b^	80.59 aC^b^	67.97 aC^b^	156.12 aB^a^	261.35 aA^b^ ^a^
BV03-PA	29.93 aC^b^	98.56 aB^b^	60.77 aB^b^	75.56 aB^b^	142.13 bA^a^
control with BV03-TS^a^	78.87				
control without nematicide and herbicide^b^	155.32				
CV	54.1				

Root dry matter (g)—60 DAE					
	[Flum. + *S*-met.]	*S*-met.	[Fomes. + *S*-met.]	[Flum. + pirox.]	[Flum. + imaz.]
without nematicide	37.25 aA^a^ ^b^	22.00 aA	29.75 aA^b^	29.50 aA^b^	19.50 aB^a^
BV03-PA	33.25 aA^b^	16.25 bB^a^ ^b^	32.00 aA^b^	24.00 bA^b^	25.25 bA^b^
control with BV03-TS^a^	25.75				
control without nematicide and herbicide^b^	17.25				
CV	20.42				

Root volume (cm^3^)—60 DAE					
	[Flum. + *S*-met.]	*S*-met.	[Fomes. + *S*-met.]	[Flum. + pirox.]	[Flum. + imaz.]
without nematicide	637.50 aA	627.50 aA	628.75 aA	632.50 aA	627.50 aA
BV03-PA	635.00 aA	620.00 aA	630.00 aA	622.50 aA	635.00 aA
control with BV03-TS^a^	628.75				
control without nematicide and herbicide^b^	618.75				
CV	1.31				

a Differences between treatments and the control with BV03-PA only (BV03 applied to the foliar part) according to Dunnett’s test, *p*-value <0.05.

b Significant difference between treatments and the control without herbicide and nematicide according to Dunnett’s test, *p*-value <0.05. Lowercase letters indicate differences among treatments in the columns, and uppercase letters indicate differences in the rows, according to the Scott-Knott test, *p*-value <0.05. CV = coefficient of variation. Flum. + *S*-met. = flumioxazin + *S*-metolachlor; *S*-met. = *S*-metolachlor; Fomes. + *S*-met. = fomesafen + *S*-metolachlor; Flum. + pyrox. = flumioxazin + pyroxasulfone; Flum. + imaz. = flumioxazin + imazethapyr.

In the presence of the nematicide BV03-PA, the number of nematodes ranged from 29.93 to 142.13, showing distinct effects among herbicide treatments (Table 2). The lowest values were observed in the treatment [flumioxazin + *S*-metolachlor] (29.93), followed by [fomesafen + *S*-metolachlor] (60.77), both lower than those of the control with BV03-PA (78.87), indicating a possible complementary action of the herbicides in reducing nematode populations. The treatments with *S*-metolachlor (98.56) and [flumioxazin + pyroxasulfone] (75.56) presented values similar to those of the control with BV03-PA, suggesting no increase or decrease in control. On the other hand, the treatment with [flumioxazin + imazethapyr] showed the highest number of nematodes (142.13), even surpassing the nematicide control, suggesting a possible negative interference of this treatment on the performance of BV03-PA in pathogen management.

In treatments without nematicide, the mean root dry mass for [flumioxazin + *S*-metolachlor] (37.25 g), [fomesafen + *S*-metolachlor] (29.75 g), [flumioxazin + pyroxasulfone] (29.50 g), and *S*-metolachlor (22.00 g) was higher than the control without herbicide and nematicide (17.25 g). The treatment [flumioxazin + *S*-metolachlor] was also superior to the control with BV03-PA. On the other hand, the lowest accumulation for this variable was observed for [flumioxazin + imazethapyr] (19.50 g), which did not differ statistically from the control without nematicide and was lower than the control with BV03-PA.

For treatments with BV03-PA application, higher root dry mass accumulation was observed for [flumioxazin + *S*-metolachlor] (33.25 g), [fomesafen + *S*-metolachlor] (32.00 g), [flumioxazin + pyroxasulfone] (24.00 g), and [flumioxazin + imazethapyr] (25.25 g) compared to the control without herbicide and nematicide (17.25 g). These treatments did not differ from those of the control with BV03-PA. *S*-metolachlor showed the lowest value (16.25 g), lower than both controls. No differences were observed among treatments for root volume (Table 2).

### Interaction between Seed Treatment Application of *Bacillus amyloliquefaciens* BV03 and Herbicides Applied in Pre-Emergence

Plant height in the absence of the nematicide ranged from 47.88 to 52.08 cm, with similar means among the herbicide treatments (Table 3). The treatments [flumioxazin + *S*-metolachlor] (52.08 cm), *S*-metolachlor (51.83 cm), [fomesafen + *S*-metolachlor] (51.43 cm), and [flumioxazin + pyroxasulfone] (50.88 cm) showed higher values than the control without herbicide and nematicide (42.00 cm), indicating a possible stimulus to initial development, even without nematode control. The treatment with [flumioxazin + imazethapyr] did not differ from that of the controls. In the presence of the nematicide BV03-ST, the highest plant height was observed with [flumioxazin + *S*-metolachlor] (55.43 cm) compared to the other herbicides, surpassing both controls (48.07 cm with BV03-ST and 42.00 cm without nematicide). The treatments with [flumioxazin + pyroxasulfone] (51.45 cm), *S*-metolachlor (49.88 cm), and [fomesafen + *S*-metolachlor] (50.00 cm) showed values similar to those of the control with BV03-ST but were higher than the control without herbicide and nematicide. The treatment [flumioxazin + imazethapyr] showed the lowest plant height compared to those of other herbicides and controls.

**Table 3:** Plant Height (cm), Stem Diameter (cm), and Number of Leaves of Soybean Plants Subjected to Seed Treatment with Nematicide (*Bacillus amyloliquefaciens* BV03–BV03-ST) and in the Presence and Absence of Herbicides Applied at Pre-Emergence

plant height (cm)—60 DAE					
	[Flum. + *S*-met.]	*S*-met.	[Fomes. + *S*-met.]	[Flum. + pirox.]	[Flum. + imaz.]
without nematicide	52.08 aA^b^	51.83 aA^b^	51.43 aA^b^	50.88 aA^b^	47.88 aA
BV03-TS	55.43 aA^a^ ^b^	49.88 aB^b^	50.00 aB^b^	51.45 aB^b^	34.40 bC^a^ ^b^
control with BV03-TS^a^	48.07				
control without nematicide and herbicide^b^	42.00				
CV	6.28				

Stem diameter (mm)—60 DAE					
	[Flum. + *S*-met.]	*S*-met.	[Fomes. + *S*-met.]	[Flum. + pirox.]	[Flum. + imaz.]
without nematicide	5.72 aA	4.89 bB	5.76 aA	5.47 aA	4.70 aB
BV03-TS	5.62 aA	5.12 aB	5.47 aA	5.42 aA	4.65 bA
control with BV03-TS^a^	4.63				
control without nematicide and herbicide^b^	4.38				
CV	6.28				

Number of leaves—60 DAE					
	[Flum. + *S*-met.]	*S*-met.	[Fomes. + *S*-met.]	[Flum. + pirox.]	[Flum. + imaz.]
without nematicide	17.00 aA^a^ ^b^	18.75 aA^a^ ^b^	13.00 aB	16.50 aA^b^	14.25 aB
BV03-TS	16.00 aA^b^	17.75 aA^a^ ^b^	13.00 aB	14.50 aB	9.00 bC^a^
control with BV03-TS^a^	13.33				
control without nematicide and herbicide^b^	12.5				
CV	12.47				

a Differences between treatments and the control with BV03-PA only (BV03 applied to the foliar part) according to Dunnett’s test, *p*-value <0.05.

b Significant difference between treatments and the control without herbicide and nematicide according to Dunnett’s test, *p*-value <0.05. Lowercase letters indicate differences among treatments in the columns, and uppercase letters indicate differences in the rows, according to the Scott-Knott test, *p*-value <0.05. CV = coefficient of variation. Flum. + *S*-met. = flumioxazin + *S*-metolachlor; *S*-met. = *S*-metolachlor; Fomes. + *S*-met. = fomesafen + *S*-metolachlor; Flum. + pyrox. = flumioxazin + pyroxasulfone; Flum. + imaz. = flumioxazin + imazethapyr.

There were no differences among treatments for the stem diameter (Table 3). In the absence of nematicide, the treatments with *S*-metolachlor (18.75) and [flumioxazin + *S*-metolachlor] (17.00) showed the highest values for number of leaves, higher than both the control without herbicide and nematicide (12.50) and the control with BV03-ST (13.33). The treatment with [flumioxazin + pyroxasulfone] (16.50) was also higher than the control without nematicide but did not differ from the control with BV03-ST. For the treatments with [flumioxazin + imazethapyr] (14.25) and [fomesafen + *S*-metolachlor] (13.00), the lowest leaf counts were observed compared to the other herbicides, with no difference in relation to the controls.

With the application of the nematicide BV03-ST (Table 3), the treatments [flumioxazin + *S*-metolachlor] and *S*-metolachlor (17.75) maintained the highest leaf numbers compared to the other treatments, which were also higher than both controls (13.33 with BV03-ST and 12.50 without nematicide). The treatment [flumioxazin + *S*-metolachlor] (16.00) also showed a higher number of leaves compared to the control without nematicide, although it did not differ from the control with BV03-ST. The treatments with [flumioxazin + pyroxasulfone] (14.50) and [fomesafen + *S*-metolachlor] (13.00) did not differ from the controls, indicating no increase or reduction. On the other hand, [flumioxazin + imazethapyr] showed the lowest value (9.00), lower than both controls.

The number of nematodes per gram of root in the absence of nematicide ranged from 23.20 to 261.35, with marked differences among the herbicide treatments (Table 4). The lowest values were observed in the treatments with *S*-metolachlor (23.20), [flumioxazin + *S*-metolachlor] (52.64), and [fomesafen + *S*-metolachlor] (67.97), all lower than those in the control without herbicide and nematicide (155.32). The treatment with [flumioxazin + pyroxasulfone] (156.12) showed a mean similar to the control without nematicide, indicating no positive effect on pathogen suppression. The treatment with [flumioxazin + imazethapyr] (261.35) resulted in the highest nematode population, exceeding both controls.

**Table 4:** Number of Nematodes per 10 g of Root (g), Root Dry Mass (g), and Root Volume (cm^3^) of Soybean Plants Subjected to Seed Treatment with Nematicide (*Bacillus amyloliquefaciens* BV03–BV03-ST) and in the Presence and Absence of Herbicides Applied at Pre-Emergence

number of nematodes per 10 g of root—60 DAE					
	[Flum. + *S*-met.]	*S*-met.	[Fomes. + *S*-met.]	[Flum. + pirox.]	[Flum. + imaz.]
without nematicide	52.64 aB^b^	23.20 bB^b^	67.97 bB^b^	156.12 aA^a^	261.35 bA^a^ ^b^
BV03-TS	77.70 aC^b^	290.09 aB^a^ ^b^	233.28 aB^a^ ^b^	30.84 bC^b^	421.90 aA^a^ ^b^
control with BV03-TS^a^	58.84				
control without nematicide and herbicide^b^	155.32				
CV	51.83				

Roots dry matter (g)—60 DAE					
	[Flum. + *S*-met.]	*S*-met.	[Fomes. + *S*-met.]	[Flum. + pirox.]	[Flum. + imaz.]
without nematicide	37.25 aA^a^ ^b^	22.00 aC	29.75 aB^b^	29.50 bB^b^	19.50 bC
BV03-TS	32.50 aA^a^ ^b^	25.75 aC	28.00 aB^b^	34.00 aA^a^ ^b^	24.75 aC
control with BV03-TS^a^	21.33				
control without nematicide and herbicide^b^	17.25				
CV	17.5				

Root volume (cm^3^)—60 DAE					
	[Flum. + *S*-met.]	*S*-met.	[Fomes. + *S*-met.]	[Flum. + pirox.]	[Flum. + imaz.]
without nematicide	637.50 aA	627.50 aA	628.75 aA	632.50 aA	627.50 aA
BV03-TS	621.25 bB	628.75 aA	622.50 Aa	635.00 aA^b^	615.00 bB
control with BV03-TS^a^	628.33				
control without nematicide and herbicide^b^	618.75				
CV	1.21				

a Indicates differences between treatments and the control with BV03-PA only (BV03 applied to the foliar part) according to Dunnett’s test, *p*-value <0.05.

b Indicates significant difference between treatments and the control without herbicide and nematicide according to Dunnett’s test, *p*-value <0.05. Lowercase letters indicate differences among treatments in the columns, and uppercase letters indicate differences in the rows, according to the Scott-Knott test, *p*-value <0.05. CV = coefficient of variation. Flum. + *S*-met. = flumioxazin + *S*-metolachlor; *S*-met. = *S*-metolachlor; Fomes. + *S*-met. = fomesafen + *S*-metolachlor; Flum. + pyrox. = flumioxazin + pyroxasulfone; Flum. + imaz. = flumioxazin + imazethapyr.

With the application of the nematicide BV03-ST, the lowest nematode count was observed in the treatment [flumioxazin + pyroxasulfone] (30.84), lower than both controls (58.84 with BV03-ST and 155.32 without herbicide and nematicide), indicating a possible complementary action of the herbicide to the nematicide’s activity. The treatment with [flumioxazin + *S*-metolachlor] (77.70) also showed satisfactory control, with a mean similar to that of the BV03-ST control and lower than that of the control without herbicide and nematicide. On the other hand, the treatments with [fomesafen + *S*-metolachlor] (233.28), *S*-metolachlor (290.09), and [flumioxazin + imazethapyr] (421.90) presented higher means compared to the other herbicides, even surpassing the controls, indicating a negative interference in the effectiveness of BV03-ST in managing nematode populations.

Greater root dry matter accumulation was observed in the treatment with [flumioxazin + *S*-metolachlor] (37.25 g) compared to the other herbicides and both controls (21.33 g with BV03-ST and 17.25 g without nematicide), suggesting that the reduction in the nematode population may have allowed for increased root growth in this treatment (Table 4). The treatments with [fomesafen + *S*-metolachlor] (29.75 g), [flumioxazin + pyroxasulfone] (29.50 g), and *S*-metolachlor (22.00 g) had higher means than the control without nematicide, likely due to the same effect observed with [flumioxazin + S-metolachlor]. The lowest value was observed for [flumioxazin + imazethapyr] (19.50 g), with no difference from the control without nematicide and lower than the control with BV03-ST, suggesting possible negative interference of high nematode population on root growth.

In the presence of nematicide BV03-ST, root dry matter ranged from 24.75 to 34.00 g, indicating variation among treatments (Table 4). The highest value was observed for [flumioxazin + pyroxasulfone] (34.00 g), also surpassing both controls. The treatment with [flumioxazin + *S*-metolachlor] (32.50 g) also resulted in greater biomass accumulation, with values higher than the controls. The treatments with [fomesafen + *S*-metolachlor] (28.00 g), *S*-metolachlor (25.75 g), and [flumioxazin + imazethapyr] (24.75 g) showed similar values, higher than the control without nematicide, but without indicating improvement over the BV03-ST control. No differences were observed for root volume in any treatment.

### Interaction between Seed Treatment Application of Fluopyram and Herbicides Applied in Pre-Emergence

Plant height in the absence of nematicide ranged from 47.88 to 52.08 cm, with similar values among the herbicide treatments (Table 5). The treatments [flumioxazin + *S*-metolachlor] (52.08 cm), *S*-metolachlor (51.83 cm), [fomesafen + *S*-metolachlor] (51.43), and [flumioxazin + pyroxasulfone] (50.88 cm) showed higher means than the control without nematicide and herbicide (42.00 cm). The treatment with [flumioxazin + imazethapyr] (47.88 cm) resulted in a height equivalent to the control without nematicide and herbicide but like the other herbicides.

**Table 5:** Plant Height (cm), Stem Diameter (cm), and Number of Leaves of Soybean Plants Subjected to Seed Treatment with the Nematicide Fluopyram and in the Presence and Absence of Herbicides Applied at Pre-Emergence

plant height (cm)—60 DAE					
	[Flum. + *S*-met.]	*S*-met.	[Fomes. + *S*-met.]	[Flum. + pirox.]	[Flum. + imaz.]
without nematicide	52.08 aA^b^	51.83 aA^b^	51.43 aA^b^	51.88 aA^b^	47.88 aA
fluopyram	51.03 aA^b^	48.13 aA	51.25 aA^b^	47.75 aA	47.68 aA
control with fluopyram^a^	48.93				
control without nematicide and herbicide^b^	42.00				
CV%	17.50				

Stem diameter (mm)—60 DAE					
	[Flum. + *S*-met.]	*S*-met.	[Fomes. + *S*-met.]	[Flum. + pirox.]	[Flum. + imaz.]
without nematicide	5.72 bA^b^	4.89 aB^b^	5.76 aA^b^	5.47 aA^b^	4.70 aB
fluopyram	6.35 aA^a^ ^b^	5.25 aA^b^	5.85 aA^b^	5.41 aB^b^	5.15 aA^b^
control with fluopyram^a^	5.35				
control without nematicide and herbicide^b^	4.38				
CV%	7.7				

Number of leaves—60 DAE					
	[Flum. + *S*-met.]	*S*-met.	[Fomes. + *S*-met.]	[Flum. + pirox.]	[Flum. + imaz.]
without nematicide	17.00 aA	18.75 aA^b^	13.00 bB	16.50 aA	14.25 bB
fluopyram	16.50 aA	18.25 aA^b^	15.50 aA	14.50 aA	16.25 aA
control with fluopyram^a^	14.00				
control without nematicide and herbicide^b^	12.50				
CV%	14.59				

a Differences between treatments and the control with BV03-PA only (BV03 applied to the foliar part) according to Dunnett’s test, *p*-value <0.05.

b Significant difference between treatments and the control without herbicide and nematicide according to Dunnett’s test, *p*-value <0.05. Lowercase letters indicate differences among treatments in the columns, and uppercase letters indicate differences in the rows, according to the Scott-Knott test, *p*-value <0.05. CV = coefficient of variation. Flum. + *S*-met. = flumioxazin + *S*-metolachlor; S-met. = *S*-metolachlor; Fomes. + *S*-met. = fomesafen + *S*-metolachlor; Flum. + pyrox. = flumioxazin + pyroxasulfone; Flum. + imaz. = flumioxazin + imazethapyr.

With the application of the nematicide fluopyram, plant heights ranged from 47.68 to 51.25 cm, showing no contrasting responses among treatments (Table 5). The treatments [flumioxazin + *S*-metolachlor] (51.03 cm), [fomesafen + *S*-metolachlor] (51.25), and *S*-metolachlor (48.13 cm) provided greater height than the control without nematicide and herbicide and were like the control with fluopyram (48.93 cm). [Flumioxazin + pyroxasulfone] (47.75 cm) and [flumioxazin + imazethapyr] (47.68 cm) also did not differ from the nematicide control and were equal to the other herbicide treatments, indicating no effect among herbicides when fluopyram was present.

In the absence of nematicide, the largest stem diameters were observed with [fomesafen + *S*-metolachlor] (5.76 mm), [flumioxazin + *S*-metolachlor] (5.72 mm), and [flumioxazin + pyroxasulfone] (5.47 mm), all higher than the control without nematicide and herbicide (4.38 mm; Table 5). The treatments with *S*-metolachlor (4.89 mm) and [flumioxazin + imazethapyr] (4.70 mm) showed values like the control without nematicide.

With the application of fluopyram nematicide, the highest stem diameter was observed with [flumioxazin + *S*-metolachlor] (6.35 mm), higher than those of both the fluopyram control (5.35 mm) and the control without nematicide. The treatments with [fomesafen + *S*-metolachlor] (5.85 mm), *S*-metolachlor (5.25 mm), and [flumioxazin + imazethapyr] (5.15 mm) presented means similar to those of the fluopyram control. Overall, all treatments showed stem diameter means higher than the control without nematicide, with only [flumioxazin + imazethapyr] in the absence of nematicide showing a value equal to the control without nematicide and herbicide (Table 5).

The number of leaves per plant in the absence of nematicide ranged from 13.00 to 18.75, with differences among herbicide treatments (Table 5). The highest values were observed for *S*-metolachlor (18.75), [flumioxazin + *S*-metolachlor] (17.00), and [flumioxazin + pyroxasulfone] (16.50), all higher than the control without nematicide and herbicide (12.50). The treatment with [flumioxazin + imazethapyr] (14.25) presented an intermediate value, while the lowest number of leaves was observed with [fomesafen + *S*-metolachlor] (13.00), although it was still higher than the control without nematicide.

In the presence of fluopyram nematicide, the number of leaves ranged from 14.50 to 18.25, showing no contrasting responses among the treatments (Table 5). The treatments with *S*-metolachlor (18.25), [flumioxazin + *S*-metolachlor] (16.50), [flumioxazin + imazethapyr] (16.25), and [fomesafen + *S*-metolachlor] (15.50) showed higher means than the fluopyram control (14.00) and the control without nematicide (12.50). The treatment with [flumioxazin + pyroxasulfone] (14.50) had a mean like the fluopyram control, indicating no increase or reduction. Overall, all treatments maintained higher means than the control without nematicide, without indicating negative interference on shoot development.

The number of nematodes per gram of root ranged from 23.20 to 261.35 in the absence of nematicide, with wide variation among treatments (Table 6). The lowest values were observed for *S*-metolachlor (23.20), [flumioxazin + *S*-metolachlor] (52.64), and [fomesafen + *S*-metolachlor] (55.33), all lower than the control without herbicide and nematicide (167.1), indicating a reduction in the nematode population even without direct chemical control. The treatment with [flumioxazin + pyroxasulfone] (156.12) showed a value similar to the control without nematicide, indicating no increase or reduction. On the other hand, [flumioxazin + imazethapyr] (261.35) resulted in the highest nematode population, surpassing both controls, suggesting a possible inoculum buildup associated with this treatment.

**Table 6:** Number of Nematodes per 10 g of Root (g), Root Dry Mass (g), and Root Volume (cm^3^) of Soybean Plants Subjected to Seed Treatment with the Nematicide Fluopyram and in the Presence and Absence of Herbicides Applied at Pre-Emergence

number of nematodes per 10 g of root—60 DAE					
	[Flum. + *S*-met.]	*S*-met.	[Fomes. + *S*-met.]	[Flum. + pirox.]	[Flum. + imaz.]
without nematicide fluopyram	52.64 aC^b^	23.20 aC^b^	55.33 aC^b^	156.12 aB^a^	261.35 aA^a^ ^b^
	40.29 aA^b^	45.22 aA^b^	75.39 aA^b^	49.79 bA^b^	75.31 bA^b^
control with fluopyram^a^	60.56				
control without nematicide and herbicide^b^	167.1				
CV	46.25				

Roots dry matter (g)—60 DAE					
	[Flum. + *S*-met.]	*S*-met.	[Fomes. + *S*-met.]	[Flum. + pirox.]	[Flum. + imaz.]
without nematicide	37.25 aA^b^	22.00 aB	29.75 bB^b^	29.50 aB^b^	19.50 aC
fluopyram	34.25 aA^b^	21.25 aC	34.75 aA^b^	28.50 aB^b^	21.00 aC
control with fluopyram^a^	27.0				
control without nematicide and herbicide^b^	17.25				
CV	17.12				

Root volume (cm^3^)—60 DAE					
	[Flum. + *S*-met.]	*S*-met.	[Fomes. + *S*-met.]	[Flum. + pirox.]	[Flum. + imaz.]
without nematicide	637.50 aA^b^	627.50 bA	628.75 aA^b^	632.50 aA^b^	627.50 aA
fluopyram	636.25 aA^b^	631.25 aA	638.75 aA^b^	631.25 aA	628.75 aA
control with fluopyram^a^	628.75				
control without nematicide and herbicide^b^	618.75				
CV	1.06				

a Differences between treatments and the control with BV03-PA only (BV03 applied to the foliar part) according to Dunnett’s test, *p*-value <0.05.

b Significant difference between treatments and the control without herbicide and nematicide according to Dunnett’s test, *p*-value <0.05. Lowercase letters indicate differences among treatments in the columns, and uppercase letters indicate differences in the rows, according to the Scott-Knott test, *p*-value <0.05. CV = coefficient of variation. Flum. + *S*-met. = flumioxazin + *S*-metolachlor; *S*-met. = *S*-metolachlor; Fomes. + *S*-met. = fomesafen + *S*-metolachlor; Flum. + pyrox. = flumioxazin + pyroxasulfone; Flum. + imaz. = flumioxazin + imazethapyr.

In the presence of the nematicide fluopyram, values for nematode numbers per 100 g of root ranged from 40.29 to 75.39, with no contrasting responses among treatments (Table 6). The treatment [flumioxazin + *S*-metolachlor] (40.29) showed a value lower than that of the fluopyram control (60.56) and the control without nematicide (167.1), indicating possible complementary action. The other treatments presented similar values (between 45.22 and 75.39), all lower than the control without nematicide but without showing a relevant increase or reduction compared with the fluopyram control.

Root dry matter ranged from 19.50 to 37.25 g in the absence of nematicide, with emphasis on the treatment [flumioxazin + *S*-metolachlor] (37.25 g), which showed the highest accumulation, greater than both the fluopyram control (27.00 g) and the control without nematicide (17.25 g), indicating possible complementary action. [Fomesafen + *S*-metolachlor] (29.75 g) and [flumioxazin + pyroxasulfone] (29.50 g) also showed higher values than the control without nematicide with a positive effect on the root system. The lowest values were observed for *S*-metolachlor (22.00 g) and [flumioxazin + imazethapyr] (19.50 g), with no difference from the control without a nematicide.

With fluopyram, root dry matter ranged from 21.00 to 34.75 g. The highest value was observed for [fomesafen + *S*-metolachlor] (34.75 g), followed by [flumioxazin + *S*-metolachlor] (34.25 g), both higher than those of the fluopyram control and the control without nematicide, suggesting possible complementary action. [Flumioxazin + pyroxasulfone] (28.50 g) also exceeded the control without nematicide with intermediate behavior. The lowest values were observed for *S*-metolachlor (21.25 g) and [flumioxazin + imazethapyr] (21.00 g), with no increases relative to the control.

Root volume showed narrow variation among treatments, with values ranging from 627.50 to 637.50 cm^3^ in the absence of a nematicide (Table 6). [Flumioxazin + *S*-metolachlor] (637.50), [fomesafen + *S*-metolachlor] (628.75 cm^3^), and [flumioxazin + pyroxasulfone] (632.50 cm^3^) showed higher values than the control without nematicide (618.75 cm^3^), indicating a slight stimulus to root volume. The treatment with *S*-metolachlor (627.50 cm^3^) showed a lower value than the others but still higher than the control.

With fluopyram, the values ranged from 628.75 to 638.75 cm^3^. The treatments [flumioxazin + *S*-metolachlor] (636.25 cm^3^), [fomesafen + *S*-metolachlor] (638.75 cm^3^), and [flumioxazin + pyroxasulfone] (631.25 cm^3^) showed higher values than the control without nematicide and were similar to the fluopyram control (628.75 cm^3^), without indicating contrasting responses. All treatments maintained higher means than the control without nematicide, without indicating negative interference with root system development.

## Discussion

Combinations between five pre-emergence herbicide treatments and three nematicide treatments were evaluated to identify the interaction of each combination regarding nematode control. In general, the combinations involving the biological nematicide BV03 showed a tendency for antagonistic effects when in the presence of pre-emergence herbicide applications, especially when biocontrol was applied via seed treatment (BV03-ST). In this way, the presence of these herbicides in the soil may negatively interfere with the survival or activity of the biological agent, reducing its effectiveness against *P. brachyurus*. Residual herbicides in the soil often alter the microbial community and can suppress beneficial organisms,^18,19^ which may explain the lower capacity of BV03 applied via seed treatment to suppress *P. brachyurus* populations. For example, the application of herbicides such as *S*-metolachlor, [flumioxazin + *S*-metolachlor], [fomesafen + *S*-metolachlor], [flumioxazin + pyroxasulfone], and [flumioxazin + imazethapyr], in the presence of BV03-ST, showed lower control compared to the control without herbicides and with nematicide, possibly due to reduced viability or colonization of the biocontrol in the rhizosphere caused by these chemical compounds.

On the other hand, for BV03 applied via foliar spray (BV03-FA), the interaction with herbicides was predominantly additive. In this modality, the control agent was applied to the leaves, reducing the direct contact with the herbicides present in the soil. This may have allowed each active ingredient (herbicide and *B. amyloliquefaciens* BV03) to act relatively independently, without interference between them due to the lack of direct contact in the soil. For BV03-FA applications, such as [flumioxazin + *S*-metolachlor], *S*-metolachlor, [fomesafen + *S*-metolachlor], and [flumioxazin + pyroxasulfone], there was no clear indication of synergism; i.e., the combined effect was close to the sum of the individual effects (additive interaction).

The effectiveness of the strategy involving the application of BV03 to the aerial part of the plants is strongly linked to the induction of systemic resistance (ISR) in the host plant. Microorganisms applied to the leaves can activate defense responses that spread systemically, conferring protection to the roots against nematodes. Studies with *Bacillus* spp. and other biocontrol agents show that they can induce systemic resistance via activation of defense genes (PR proteins) in plants, reducing nematode penetration and development in the roots.^20^ In the case of soybean, a *Bacillus halotolerans* isolate applied to the plants was able to increase the expression of defense genes related to the salicylic acid and jasmonic acid pathways and, with this, suppress cyst nematode infection in the root system.^20^ Consequently, a lower initial exposure to the herbicide may avoid the direct antagonistic effect of herbicides on the microorganism, making *B. amyloliquefaciens* BV03 potentially more compatible with the herbicides used.

The combinations with the nematicide fluopyram also resulted in additive effects, with no evidence of pronounced synergism or antagonism. In all tested combinations (fluopyram and each of the five herbicide treatments), the nematode control obtained was approximately what was expected from the sum of the action of each product individually. Laboratory studies support this observation: for example, in the combination of chemical nematicides with herbicides, there was no reduction in the nematicide effect due to the presence of the herbicide.^13^ In the present case, herbicides did not negatively interfere with the effectiveness of fluopyram, maintaining control at a level equivalent to that of the isolated nematicide.

The patterns of synergism, antagonism, or absence of interaction observed for the treatments tested here can be explained by the underlying mechanisms involving plant physiology, soil microbiota, and product chemistry. Antagonistic effects, such as those observed between pre-emergence herbicides and the biological nematicide BV03, generally result from the impact of herbicides on microorganisms. Many residual herbicides can alter the composition and activity of the microbiota, either through direct toxicity to certain microbial groups or indirectly through modification of the rhizospheric environment. Meta-analyses indicate that herbicide application can reduce the abundance of predatory and fungivorous nematodes (beneficial organisms that naturally help control nematodes), while increasing the abundance of plant-parasitic nematodes.^21^ This suggests that herbicides can reduce the microbial community of competitor organisms or natural enemies of nematodes, favoring their proliferation.

For BV03, herbicides such as *S*-metolachlor, [flumioxazin + *S*-metolachlor], [fomesafen + *S*-metolachlor], [flumioxazin + pyroxasulfone], and [flumioxazin + imazethapyr], especially under conditions where BV03 was applied via seed treatment, may have inhibited the growth, survival, or function of this microorganism, resulting in reduced control. For example, *S*-metolachlor and other acetanilide herbicides are known to affect soil microorganisms, reducing soil microbial biodiversity by suppressing beneficial bacteria, including some associated with nitrogen fixation.^22^ This may explain the lower efficacy of BV03-ST in combination with *S*-metolachlor or mixtures containing this active ingredient as root colonization by the biological agent would be compromised. Similarly, ALS-inhibiting herbicides (such as imazethapyr) and PPO inhibitors (flumioxazin and fomesafen) can cause oxidative stress or interfere with the metabolism of soil fungi and bacteria,^23,24^ possibly making the environment less favorable for *B. amyloliquefaciens* BV03. This would explain the reduction in nematicide control under conditions where the [flumioxazin + imazethapyr] mixture was applied to the soil.

In contrast, fluopyram, a nematicide with a specific mode of action (inhibits the enzyme succinate dehydrogenase in nematodes, paralyzing their cellular respiration),^25,26^ did not show negative interactions with most tested herbicides. Its greater mode-of-action specificity probably makes it more selective and less prone to interference from other chemical compounds, such as herbicides. This finding corroborates previous results in which herbicides, such as metribuzin or trifluralin, did not reduce the efficacy of organophosphate or carbamate nematicides. Moreover, laboratory studies showed inhibition of nematode egg hatching primarily due to the nematicide (e.g., fenamiphos), without herbicide addition impairing this effect.^13^ Another important point is the chemical interactions in the soil. There was no indication that the herbicides stimulated the degradation or inactivation of fluopyram.

Some herbicides showed potential to suppress *P. brachyurus* populations, such as [flumioxazin + *S*-metolachlor], *S*-metolachlor, and [fomesafen + *S*-metolachlor], even in the absence of a nematicide, whether chemical or biological. Several factors may explain this effect of herbicides on *P. brachyurus* population suppression, even without direct action of these herbicides on nematode metabolic or physiological pathways. For example, herbicides may promote the growth of certain bacteria and fungi naturally present in the soil and capable of suppressing *P. brachyurus* populations. In addition, changes in the root microbiome and rhizospheric environment due to the presence of these herbicides may also alter root exudate composition, which in turn can make soybean roots attractive to nematodes.^27^

Nematode pressure on plants had a direct effect on soybean crop vigor and initial development. In general, greater nematode control was associated with better vegetative performance, whereas insufficient control led to reduced growth, especially in root biomass accumulation. In fields infested with *P. brachyurus*, it is common to observe patches of stunted and yellowish plants.^28^ These symptoms result from nematode feeding on the roots, which destroy vascular tissues, cause lesions, and impair the plant’s water and nutrient uptake. Consequently, plants under high nematode pressure show reduced height, thinner stems, and fewer leaves compared with healthy plants.

In the present study, this relationship was confirmed; i.e., treatments that provided better suppression of the nematode population allowed soybean plants to grow and develop better, whereas in treatments where nematodes were not well controlled, plants showed lower root biomass accumulation, fewer leaves, and smaller stem diameter. In treatments that were effective in suppressing *P. brachyurus* populations—such as successful combinations of herbicides with fluopyram or BV03—average plant height and root dry mass were higher, indicating that nematode elimination/reduction allowed soybeans to express their initial growth potential. For treatments with low control, such as [flumioxazin + imazethapyr], the plants remained smaller. This difference is confirmed in the literature: In controlled experiments, nematode-free soybean plants grow significantly more than infected plants.^28^ Studies show that under heavy infestation, soybeans often abort leaves or stop producing new leaves at the expected rate due to altered hormonal balance and nutrient limitation caused by nematodes.

The effectiveness of *P. brachyurus* management in soybean cultivation directly depends on the compatibility between pre-emergence herbicides and the nematicides used, whether biological or chemical. The application of the biological nematicide *Bacillus amyloliquefaciens* BV03 proved to be more effective when applied to the foliar part of the plant, showing predominant additive interactions with herbicides, especially in treatments such as [flumioxazin + *S*-metolachlor] and [fomesafen + *S*-metolachlor]. On the other hand, seed treatment with *B. amyloliquefaciens* BV03 was less effective in the presence of certain herbicides under the tested conditions, indicating a possible antagonistic effect.

Fluopyram application exhibits high selectivity with effective control of the nematode population even in the presence of herbicides applied at pre-emergence. In addition, some herbicides, such as *S*-metolachlor and its combinations with flumioxazin or fomesafen, demonstrated a suppressive effect on *P. brachyurus* populations, even in the absence of nematicides.

## Conclusions

[Flumioxazin + imazethapyr] combination increased nematode populations, whereas [flumioxazin + *S*-metolachlor] and [fomesafen + *S*-metolachlor] reduced nematode populations and promoted soybean root biomass accumulation. The [flumioxazin + imazethapyr] mixture also impaired the nematicidal activity of *B. amyloliquefaciens* BV03, both as a seed treatment and as a foliar spray. In contrast, the antagonistic effect of [flumioxazin + pyroxasulfone] on *B. amyloliquefaciens* BV03 was detected only when the nematicide was applied as a seed treatment.

Some herbicides suppressed *P. brachyurus* populations independently of nematicide application, while others exhibited additive effects when combined with the nematicide. Overall, the interactions observed in this study highlight the need for careful product selection within an integrated management framework, considering not only the efficacy of each input individually but also their combined impacts on soil and plant health.

## Supplementary Material



## Notes

(Table S1) Physicochemical properties of the active ingredients evaluated in this study, including molecular structure, CAS registry number, vapor pressure, and water solubility at 20 °C (PDF)

## Abbreviations

DAA: application
TRT: treatment
DC: double check
