# Supplementary material for: Interactions between Pre-Emergence Herbicides and Nematicides on the Soybean Growth and Nematode Population
Source: ACS Omega. 2025 Dec 4;10(49):60786–97. doi: 10.1021/acsomega.5c08857 (PMC12713441; doi:10.1021/acsomega.5c08857)
Supplement: Supplementary file 1 [file ao5c08857_si_001.pdf]

Interactions between pre-emergence herbicides and nematicides on the soybean growth and nematode population

João Guilherme Queiroz Bordignon[1], Pedro Delefrate Neto[2], Hércules Diniz Campos[3], Laís Tereza Rego Torquato Reginaldo[4], Camila Rebelatto Muniz[1], Guilherme Braga Pereira Braz[3]*, Matheus de Freitas Souza[3]

[1] Master Degree student – Plant Production Graduate Program, Universidade de Rio Verde, Rio Verde, Brazil.

[2] Undergrad student – Agronomy College, Universidade de Rio Verde, Rio Verde, Brazil.

[3] Professor – Plant Production Graduate Program, Universidade de Rio Verde, Rio Verde, Brazil.

[4] Staff, Mississippi State University, Starkville, United States of America.

[*] *Corresponding author. Tel.: +55 64 3620-3013. E-mail address: guilhermebrag@gmail.com*

Supplemetary files

Table S1. Physicochemical properties of the active ingredients evaluated in this study, including molecular structure, CAS registry number, vapor pressure, and water solubility at 20 °C.

Compound	CAS number	Molecular formula	Vapor pressure (mm Hg)	Solubility in water (mg L^{-1} at 20 °C)
S-metolachlor	87392-12-9	$C_{15}H_{22}ClNO_2$	1.3 x 10^{-6}	480
Fomesafen	72178-02-0	$C_{15}H_{10}ClF_3N_2O_6S$	8 x 10^{-8}	~ 50
Imazethapyr	81335-77-5	$C_{15}H_{19}N_3O_3$	1 x 10^{-7}	1400
Pyroxasulfone	447399-55-5	$C_{12}H_{14}F_5N_3O_4S$	1.8 x 10^{-8}	3.49
Fluopyram	658066-35-4	$C_{16}H_{11}ClF_6N_2O$	9 x 10^{-9}	16

*Physicochemical parameters (CAS number, molecular formula, vapor pressure, and water solubility) were retrieved from PubChem (https://pubchem.ncbi.nlm.nih.gov) and the Pesticide Properties DataBase (PPDB; https://sitem.herts.ac.uk/aeru/ppdb/).
